# Integrating AI with PCR for Tuberculosis Diagnosis: Evaluating a Deep Learning Model for Chest X-Rays

**DOI:** 10.3390/bioengineering12121377

**Published:** 2025-12-18

**Authors:** Wei-Cheng Chiu, Shan-Yueh Chang, Chin Lin, Teng-Wei Chen, Wen-Hui Fang

**Affiliations:** 1 Department of Family and Community Medicine, Tri-Service General Hospital, National Defense Medical University, Taipei 114, Taiwan, China; chiuchen5@gmail.com; 2 Division of Pulmonary and Critical Care Medicine, Department of Internal Medicine, School of Medicine, Tri-Service General Hospital, National Defense Medical University, Taipei 114, Taiwan, China; leehornwok@gmail.com; 3 Graduate Institute of Life Sciences, National Defense Medical University, Taipei 114, Taiwan, China; xup6fup0629@gmail.com; 4 Medical Technology Education Center, School of Medicine, National Defense Medical University, Taipei 114, Taiwan, China; 5 Department of Artificial Intelligence and Internet of Things, Tri-Service General Hospital, National Defense Medical University, Taipei 114, Taiwan, China; 6 School of Public Health, National Defense Medical University, Taipei 114, Taiwan, China; 7 Division of General Surgery, Department of Surgery, Tri-Service General Hospital, National Defense Medical University, Taipei 114, Taiwan, China; doc20264@gmail.com; 8 Artificial Intelligence of Things Center, Tri-Service General Hospital, National Defense Medical University, Taipei 114, Taiwan, China

**Keywords:** tuberculosis, deep learning, chest X-rays, polymerase chain reaction

## Abstract

Tuberculosis (TB) remains a major global health challenge, and early, accurate diagnosis is essential for effective disease control. Chest radiography (CXR) is widely used for TB screening because of its accessibility, yet its limited specificity necessitates confirmatory molecular testing such as polymerase chain reaction (PCR) assays. This study aimed to evaluate the diagnostic performance of a deep learning model (DLM) for TB detection using CXR and to compare its predictive accuracy with PCR results, specifically in a low-burden region. A retrospective dataset of CXR images and corresponding PCR findings was obtained from two hospitals. The DLM, based on the CheXzero vision transformer, was trained on a large imaging dataset and evaluated using receiver operating characteristic (ROC) curves and area under the curve (AUC) metrics. Internal and external validation sets assessed sensitivity, specificity, and predictive values, with subgroup analyses according to imaging modality, demographics, and comorbidities. The model achieved an AUC of 0.915 internally and 0.850 externally, maintaining good sensitivity and specificity, though performance declined when limited to PCR-confirmed cases. Accuracy was lower for older adults and those with chronic kidney disease, chronic obstructive pulmonary disease, or heart failure. These findings suggest AI-assisted CXR screening may support TB detection in resource-limited settings, but PCR confirmation remains essential.

## 1. Introduction

Tuberculosis (TB), a chronic infectious disease caused by Mycobacterium tuberculosis, predominantly targets the lungs [1]. Tuberculosis poses a significant global public health challenge. In 2023, approximately 10.8 million individuals developed TB, representing a modest increase from the 10.7 million cases reported in 2022. The disease also claimed approximately 1.25 million lives in 2023. With COVID-19 deaths declining, it is likely that TB has once again become the leading global cause of death via a single infectious pathogen [2]. However, delays in the diagnosis and treatment of pulmonary TB present a significant obstacle to global TB control initiatives and can have detrimental consequences for both individuals and public health [3]. Bello et al. conducted a systemic review to estimate average TB diagnosis delay (the time lapse between the onset of symptoms and the initiation of treatment) and reported that the pooled mean total delay was 87.6 days [4]. Early identification of TB is crucial for prompt initiation of treatment [5], which not only improves patient outcomes [6] but also helps prevent the spread of the disease within communities [7].

Chest radiography (CXR), or a chest X-ray, is commonly used as a screening tool for detecting TB disease due to its sensitivity across all populations [8]. However, CXR alone lacks the specificity required to confirm a TB diagnosis. Its use in TB screening and triage is further constrained by the limited availability of trained healthcare professionals that can interpret radiographic images as well as the significant intra- and inter-reader variability in detecting TB-related abnormalities. As a result, CXR-based screening is often followed by a confirmatory diagnostic test to ensure accurate detection and diagnosis [9].

One of the most widely used diagnostic methods for TB is the polymerase chain reaction (PCR)-based test, particularly the Xpert MTB/RIF assay [10,11], which enables rapid detection of M. tuberculosis. The COVID-19 pandemic accelerated advancements in PCR technology [12], enhancing multiplex PCR techniques that allow for the simultaneous detection of multiple pathogens with improved sensitivity and specificity. These innovations have now been integrated into TB diagnostics, improving accessibility in TB-endemic regions [13]. However, despite these advances, PCR-based methods remain costly and logistically demanding, making them less practical for large-scale screening in resource-limited settings and highlighting the need for complementary diagnostic strategies.

Recent years have seen substantial progress in applying artificial intelligence (AI), particularly deep learning and machine learning, to disease detection across diverse clinical domains. Transfer-learning-enhanced convolutional neural networks (CNNs) have enabled computationally efficient multi-cancer detection, even with limited training data [14]. Low-complexity CNN architectures have also demonstrated strong performance in tumor anomaly identification and brain tumor classification [15,16]. In population-level applications, improved recurrent neural network variants have been shown to boost epidemic forecasting accuracy and support timely public health responses [17]. In cardiovascular medicine, recent systematic reviews highlight the expanding role of deep learning in heart failure detection, risk stratification, and clinical decision support [18]. Advances in uncertainty-aware CNNs have further strengthened medical image classification by improving model reliability and robustness [19]—an important consideration in safety-critical diagnostic workflows. Recent work has also demonstrated the effectiveness of deep learning with respect to chest radiographs for pulmonary disease and TB screening. A Heliyon 2024 study introduced a deep learning pipeline for pulmonary abnormality detection from CXR images, reaffirming the clinical relevance of imaging-based AI for respiratory disease assessment [20]. In parallel, Transformer-based architectures have gained increasing prominence in medical imaging. Vision Transformer (ViT) frameworks—including contrastive learning models such as PASTER—have shown strong capability in capturing high-level semantic representations from radiographs [21]. Hybrid architectures combining Transformer-derived embeddings with gradient-boosted decision models, such as the ViT–XGBoost system reported in *Neurocomputing 2024*, provide methodological support for our approach of using ViT features with a lightweight classifier [22]. Collectively, these advances illustrate the rapid evolution of AI-driven methodologies in disease detection and provide an essential foundation for applying deep learning models to automated TB screening [23].

Recent AI-driven CXR interpretation tools such as CAD4TB, qXR, and Lunit INSIGHT [24,25,26] have demonstrated strong performance in TB detection, with most evaluations conducted in high-burden settings across Asia and Africa, where sputum PCR samples and large-scale screening cohorts are readily available. However, evidence from low-burden regions remains limited. TB prevalence in high-burden countries of South/Southeast Asia and sub-Saharan Africa ranges from 200 to over 900 per 100,000, while Taiwan’s prevalence is about 62 per 100,000, illustrating a marked disparity in disease burden [27,28,29]. Despite the fact that disease prevalence substantially influences model performance, particularly positive and negative predictive values, this gap is clinically important, as AI systems optimized for high-prevalence environments may not generalize reliably to settings where TB is infrequent. To address this, our study focuses on evaluating a deep learning model in Taiwan—a low-burden region—to investigate its predictive performance when integrated with PCR confirmation. Our goal is to provide complementary evidence to existing CAD frameworks and help clarify the utility of AI-assisted CXR screening in low-prevalence populations by directly integrating PCR results as a reference standard, which strengthens diagnostic reliability and aligns with best practices for confirmatory testing in TB screening programs.

## 2. Materials and Methods

### 2.1. Data Source and Collection

The institutional ethics committee of the Tri-Service General Hospital reviewed and approved this research (C202305019). We performed a retrospective analysis aimed at developing a deep learning model (DLM) and validating its performance both internally and externally. The dataset included CXRs acquired from June 2016 to February 2022 at two hospitals within the Tri-Service General Hospital System: an academic hospital in Neihu District (hospital A) and a community hospital in Zhongzheng District (hospital B). Chest radiographs from Hospitals A and B were obtained using different X-ray platforms to reflect real-world variability in imaging environments. Hospital A utilized CANON/TOSHIBA systems, including MRAD-A80S or MRAD-A50S generators paired with CXDI-40EG digital detectors. Hospital B employed PHILIPS + FUJIFILM configurations (BUCKY DIAGHOST TH or BUCKY DIAGNOST generators with CR-IR346RU detectors) as well as PHILIPS + AGFA systems (BUCKY DIAGHOST TH or BUCKY DIAGNOST with ADC COMPACT PLUS or CR75.0 detectors). Despite differences in hardware, all examinations were performed by licensed radiologic technologists following standardized acquisition protocols. This heterogeneity allowed us to assess model robustness and generalizability across diverse imaging systems.

We retrieved all TB complex polymerase chain reaction (PCR) test results from two hospitals between 2016 and 2022. We used the Xpert MTB/RIF (Cepheid, Sunnyvale, CA, USA) assay [30] to detect M. tuberculosis. Subsequently, based on the test dates, we identified all CXRs conducted within a positive or negative 30-day window. Following this, we categorized each individually matched CXR according to its corresponding PCR result, designating them as either positive or negative. Any remaining CXRs that did not have a matching PCR result were considered negative in the analysis.

**Figure 1:**
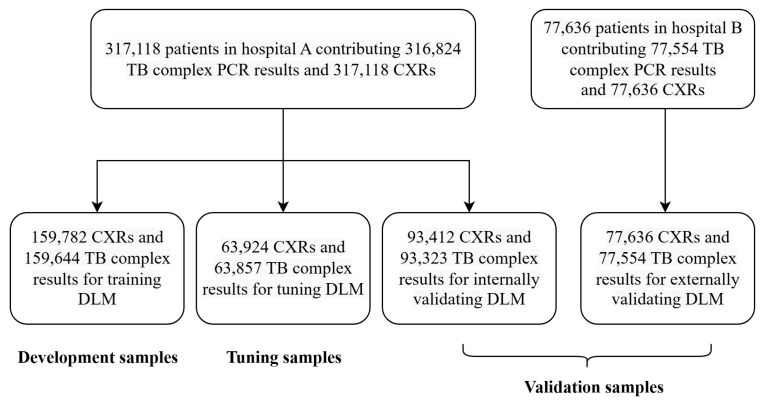
Generation of Training, Tuning, and Validation Datasets.

As shown in Figure 1, we designed the following methods for the development and validation of the DLM model. In hospital A, a total of 317,118 patients were included in this study, contributing a combined dataset of 316,824 TB complex PCR results and 317,118 CXRs. Among them, 159,782 patients were randomly assigned to the development set, providing 159,644 TB complex PCR results and 159,782 CXR records for the training of the DLM. Additionally, 63,924 patients were randomly assigned to the tuning set, contributing 63,857 TB complex PCR results and 63,924 CXR records to guide the tuning process and select the critical operating point for diagnosis. Finally, 93,412 patients were randomly assigned to the internal validation set, providing TB complex PCR results and chest X-ray records for accuracy testing and subsequent analysis. To assess the external validity of the DLM, we collected data from hospital B, including 77,636 patients who met the same inclusion criteria as those in hospital A, forming the external validation set.

We also further stratified each datasets based on the various characteristics related to CXR positioning, modality, view, gender, age, and different comorbidities including diabetes mellitus (DM), hypertension (HTN), hyperlipidemia (HLP), chronic kidney disease (CKD), heart failure (HF), coronary artery disease (CAD), and chronic obstructive pulmonary disease (COPD). Disease histories were documented according to the International Classification of Diseases, Ninth and Tenth Revisions (ICD-9 and ICD-10, respectively) from the participants’ electronic medical records. These included DM (ICD-9 codes 250.x; ICD-10 codes E11.x), HTN (ICD-9 codes 401.x–404.x; ICD-10 codes I10.x–I16.x), HLP (ICD-9 codes 272.x; ICD-10 codes E78.x), CKD (ICD-9 codes 585.x; ICD-10 codes N18.x), CAD (ICD-9 codes 410.x–414.x, 429.2; ICD-10 codes I20.x–I25.x), and COPD (ICD-9 codes 490.x–496.x; ICD-10 code J44.9).

### 2.2. Model Development

The deep learning model developed for TB prediction used high-resolution CXR images in DICOM format, each exceeding 2000 × 2000 pixels. The architecture was influenced by the CheXzero framework, which employs contrastive self-supervised learning to align CXR images with their corresponding radiology reports [31]. This alignment allows for zero-shot multi-label classification, making it possible to detect abnormalities not explicitly annotated during training [32]. By relying on natural language supervision from radiology reports instead of manually labeled datasets, the model demonstrated a high degree of generalizability across diverse clinical settings.

The core feature extraction pipeline was powered by a Vision Transformer (ViT-B/32), chosen for its capability to model long-range dependencies in medical images, which are crucial for detecting subtle TB-related anomalies [33]. CXR images were uniformly resized to 256 × 256 pixels and encoded into 512-dimensional embeddings using pretrained weights (“best_64_0.0001_original_35000_0.864.pt”), which remained unaltered throughout the training process. All original chest radiograph DICOMs (≥2000 × 2000 pixels) were resized to 256 × 256 for both training and inference due to GPU memory and throughput constraints. Central cropping was used at inference to maintain a consistent field of view. On an NVIDIA A100 GPU, feature extraction using the 512-dimensional encoder requires approximately 0.01 s per image, and the subsequent logistic regression inference adds another 0.01 s. This lightweight architecture enables near–real-time processing and makes the model feasible for high-throughput screening scenarios. No fine-tuning was applied to this image encoder. During training, images were randomly cropped to 256 × 256 without horizontal flipping, while inference used the central crop to maintain consistency [21]. And to our knowledge, the PASTER methodology does not incorporate lung-field segmentation, contrast normalization, or specialized augmentation beyond standard image resizing/cropping [21]. As a result, we followed the same preprocessing protocol in our work and did not apply segmentation or advanced normalization methods.

Model optimization was performed using the stochastic gradient descent (SGD) optimizer with a batch size of 64, a learning rate of 0.0001, and a momentum of 0.9. Weight decay (10^−4^) was applied to mitigate overfitting. All model development and training were implemented using Python version 3.10.10 and the torch library version 2.0.1.

For the classification task, the 512-dimensional embeddings were input into a logistic regression model, referred to as the CXR-risk score, to predict active TB. To benchmark the performance and provide a comparative framework, an XGBoost (eXtreme Gradient Boosting) classifier was also developed. Trained using the same dataset in R (xgboost version 0.71.2), the XGBoost model was selected for its efficiency in handling structured medical data and tabular features [34,35]. It integrated patient demographic information alongside deep learning outputs, leveraging its built-in regularization capabilities to enhance generalizability and reduce overfitting. The comparative evaluation of predictions from both models revealed that incorporating patient characteristics with image-derived features significantly improved diagnostic accuracy.

### 2.3. Statistical Analysis

The receiver operating characteristic (ROC) curve and area under the curve (AUC) were employed to assess model performance. In the tuning set, the operating point, chosen based on the maximum value of Youden’s index for detecting TB, was used for both internal and external validation. This common operating point facilitated the calculation of corresponding sensitivity, specificity, positive predictive value, and negative predictive value. Cox proportional hazards models with multiple covariates were used in the final analysis to explore associations between baseline characteristics and the key outcomes. Hazard ratios (HRs) and 95% confidence intervals (95% CIs) were employed in comparisons. Statistical analysis was conducted using R software version 3.4.4, maintaining a significance level of *p* < 0.05 throughout the entire analysis. Moreover, we conducted another analysis using only CXRs that had corresponding PCR results to assess discrepancies in our model’s performance.

## 3. Results

### 3.1. Baseline Characteristics

Patient characteristics and variables including CXR positioning, modality, view, gender, age, and different comorbidities across the training, tuning, internal validation and external validation cohorts are shown in Table 1. In the development set, 89 patients (0.1%) had TB confirmed by M. tuberculosis complex polymerase chain reaction (PCR). In the internal validation cohort, 50 patients (0.1%) had TB, while in the external validation cohort, 35 patients (0.0%) had TB. The demographic characteristics, stratified by tuberculosis diagnosis in the internal and external validation sets, are shown in Table 2.

### 3.2. Performance of CXR-TB to Identify Tuberculosis

**Figure 2:**
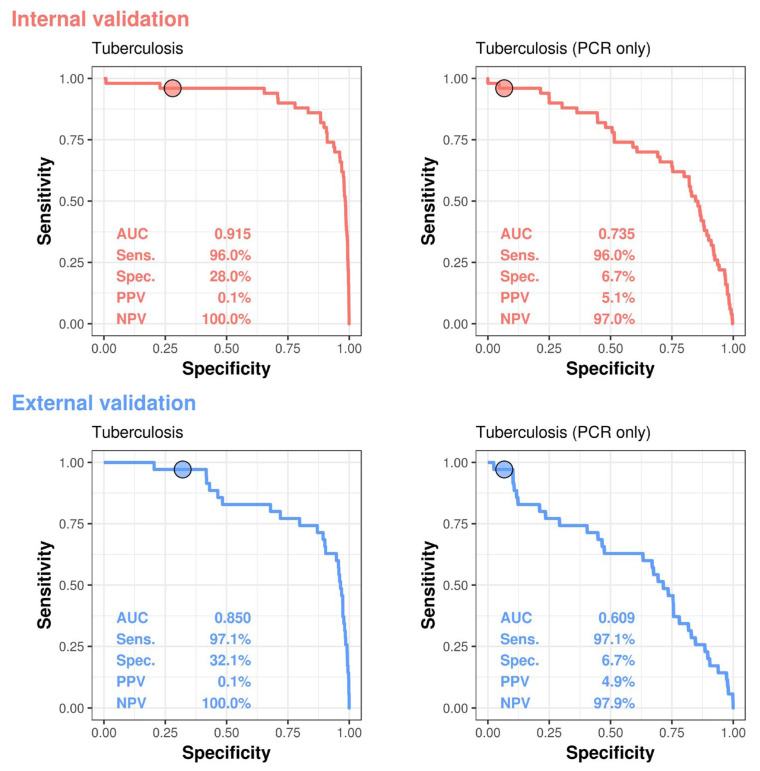
AI Model Performance for TB Detection—ROC Analysis and PCR Comparison.

All patients diagnosed with TB had an accompanying positive TB PCR report. However, for those not diagnosed with TB, we further divided the cases into two groups. The first group consisted of individuals who either had a negative PCR result or had not undergone PCR testing; these cases were classified as TB-negative. The second group (PCR-only) included cases that were only classified as TB-negative if a corresponding negative PCR report was available. Figure 2 provides analyses of diagnostic tests for TB, comparing differences in performance between internal and external validations, and between general and PCR-only tests. Deep learning model demonstrated the area under the curve (AUC) for detecting TB, with an AUC of 0.915 (95% confidence interval, CI: 0.864–0.967) and corresponding sensitivity of 86.0%, specificity of 88.1%, positive predictive value of 0.4%, and negative predictive value of 100% in the internal validation set; and an AUC of 0.850 (95% CI: 0.776–0.924) and corresponding sensitivity of 71.4%, specificity of 87.6%, positive predictive value of 0.3%, and negative predictive value of 100% in the external validation set. For analysis only with CXRs that had corresponding PCR results, the AUC for identifying TB was 0.735 (95% CI: 0.658–0.811; sensitivity 86.0%, specificity 44.3%) in the internal validation set and 0.609 (95% CI: 0.502–0.716; sensitivity 71.4%, specificity 41.9%) in the external validation set.

**Figure 3:**
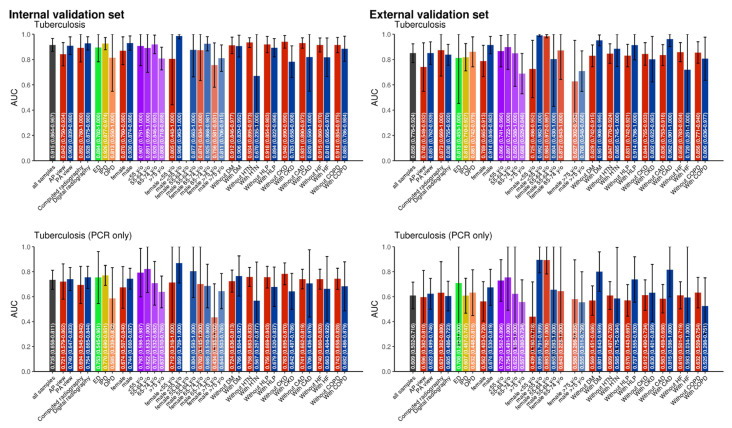
Stratified Performance of the AI Model Across Imaging Modalities, Demographics, and Comorbidities.

Model performance was further stratified by CXR views (anterior-posterior view and posterior-anterior view), CXR modalities (computed radiography and digital radiography), hospital departments (emergency department, in-patient department and out-patient department), sex (female and male), age groups (<55, 55–64, 65–74, and >75 years), comorbidities (DM, HTN, HLP, CKD, CAD, HF, and COPD) to compare the test’s performance under various conditions, as shown in Figure 3. The confidential intervals of AUCs overlap in different subgroups, which may indicate that the model performs consistently across various subgroups, suggesting a similar predictive capability for different populations or conditions.

Additionally, as shown in Supplementary Tables S1 and S2, DeLong’s test was used to compare the AUCs between the internal and external validation datasets. For the overall cohort, the AUC difference was not statistically significant (*p* = 0.153), and most subgroups likewise showed non-significant *p*-values, indicating generally consistent discrimination performance across hospitals. A few subgroups—particularly within the PCR-only analysis—demonstrated significant AUC differences, likely reflecting small sample sizes and the limited number of TB-positive cases in these strata. Overall, the results suggest that the model maintains acceptable cross-hospital generalizability, with only isolated subgroups showing variability attributable to sparse data rather than systematic performance drift.

## 4. Discussion

In this study, we developed a deep learning model (DLM) to detect TB based on CXR, which achieved an area under the curve (AUC) of 0.915 with corresponding sensitivity of 86.0% and specificity of 88.1% in the internal validation set, and an AUC of 0.850 with corresponding sensitivity of 71.4% and specificity of 87.6% in the external validation set. Currently, automated CXR analysis of various diseases including TB using DLMs is an area of significant research interest. However, upon reviewing current studies examining the performance of artificial intelligence in detecting pulmonary TB, a significant portion focuses on comparisons with radiologists. Among the various methods for diagnosing TB, only a small number of studies explicitly mention the specific diagnostic standard used. Studies that employ a positive TB polymerase chain reaction (PCR) result as the diagnostic standard are even rarer [36,37], and our study is one of the few that does so.

The results of our study, which evaluated a deep learning model for tuberculosis detection using chest X-rays and PCR validation, are comparable to those reported in recent literature. Most contemporary deep learning models for TB detection, including CAD4TB, qXR, and Lunit INSIGHT, demonstrate area under the curve (AUC) values ranging from 0.81 to 0.94, with sensitivities and specificities typically between 71% and 95% when validated against microbiological standards such as PCR or Xpert MTB/RIF [38,39]. The AUCs, sensitivities, and specificities reported in our study fall within these ranges, indicating similar diagnostic accuracy.

Recent meta-analyses and systematic reviews confirm that deep learning models consistently achieve high performance for TB detection on chest radiographs, with pooled AUCs around 0.91–0.92 and robust sensitivity and specificity [40]. The use of PCR as a reference standard in this study is consistent with best practices in the field, and our model’s performance across subgroups is in line with findings that deep learning systems maintain accuracy across diverse populations and clinical settings [38,39].

We further analyzed the performance of our DLM in predicting TB using only CXR with corresponding PCR results. The area under the ROC curve for TB detection was 0.735 (sensitivity 86.0%, specificity 44.3%) in the internal validation set, and 0.609 (sensitivity 71.4%, specificity 41.9%) in the external validation set. Our DLM demonstrated predictive performance comparable to other studies [40].

Additionally, our DLM exhibited better predictive performance in the general group compared to the PCR-only group. We hypothesize that this arises due to the fact that, in TB-negative CXRs, if a corresponding PCR test was ordered by a clinician, it is possible that non-TB lesions, which appeared suspicious for TB on the CXR, prompted the test. These non-TB lesions may have interfered with or impacted the accuracy of our DLM in predicting TB. This highlights the importance of integrating AI-based screening with molecular confirmation to optimize TB diagnosis and reduce misclassification while leveraging the strengths of both approaches.

Furthermore, as shown in Figure 3, model performance was stratified by CXR views, imaging modalities, hospital departments, sex, age groups, and comorbidities to assess its performance under different conditions. Our results indicate that while the DLM performs consistently across most subgroups, there is some variability, particularly in older adults (>75 years) and patients with chronic conditions such as CKD, COPD and heart failure, where the confidence intervals were wider, suggesting reduced certainty in these populations. Despite these variations, the model maintains strong performance across diverse settings, reinforcing its potential as a broadly applicable screening tool for TB. However, further validation is warranted in populations with significant comorbidities to ensure optimal diagnostic reliability.

The COVID-19 pandemic accelerated advancements in PCR technology, particularly in multiplex PCR [12] assays that enable simultaneous pathogen detection with improved sensitivity and specificity. These innovations have been adapted to TB diagnostics, making PCR-based methods, such as the Xpert MTB/RIF assay [10], more accessible in endemic regions. In our study, we developed a deep learning model to detect TB from chest radiographs, achieving high predictive accuracy. Notably, our AI model was benchmarked against PCR results, demonstrating its potential as a complementary screening tool. Given the model’s very high NPV (100% in the general cohort), one practical application would be its use as an initial triage tool in rural clinics or mobile chest X-ray units. In such settings, the AI system could reliably rule out TB among low-risk individuals, thereby reducing unnecessary PCR testing. For those flagged as potentially positive, the system could guide staff to prioritize proper sputum collection and refer patients for confirmatory molecular testing. This integrated approach could improve diagnostic accuracy and optimize healthcare resource allocation. Our findings also highlight that the AI model performs comparably to other deep learning-based TB detection studies [36,37,40], reinforcing its viability in clinical settings. By integrating AI-driven CXR analysis with PCR confirmation, we propose a more efficient workflow for TB diagnosis.

Our study, however, has some limitations. First, variations in image quality, acquisition protocols, and population demographics across datasets can affect the performance and generalizability of AI models [41]. Although CXRs were collected from a medical center, prospective studies are needed to validate the accuracy and applicability of our TB-detecting model in community settings. Second, the varied presentations of TB on CXRs make it difficult for our AI model to learn and generalize accurately across different cases [42,43]. A key limitation of our study is the relatively small number of TB PCR-positive samples, which may introduce class imbalance and affect the model’s ability to generalize to diverse TB cases. This imbalance could lead to an overestimation of specificity while potentially reducing sensitivity in detecting TB. Future studies with larger, more diverse TB-positive cohorts are necessary to validate our model’s predictive accuracy and enhance its robustness across different populations. Third, understanding why a DL model makes a particular decision remains a challenge. The “black-box” nature of some AI systems can hinder their acceptance in clinical practice [37,44]. However, the baseline PaS/Ter architecture used in this study was designed primarily for high-level feature extraction and does not incorporate built-in interpretability tools such as Grad-CAM or ViT attention maps. As a result, we were unable to generate these explainability visualizations for the current analysis. Methods to make AI decisions more transparent and understandable are being developed to enhance trust and clinical applicability [45,46].

Moreover, in the present study, we selected the operating point based on the maximum Youden’s index, which identifies the threshold that maximizes the sum of sensitivity and specificity. This approach provides a balanced cut-off for general diagnostic use. However, in real-world TB screening—where higher sensitivity is often prioritized—we acknowledge that alternative thresholds may be more appropriate. For instance, a secondary “high-risk” threshold can be defined at the point where sensitivity and positive predictive value are jointly maximized, allowing the system to flag individuals who warrant confirmatory PCR testing. Finally, a limitation of our implementation is the down-sampling of high-resolution DICOM images to 256 × 256, which may reduce sensitivity to small or apical lesions. For the same reason, we did not assess higher input resolutions (512–1024), multi-crop/tiling inference, or the impact of horizontal flips. We avoided horizontal flipping because laterality is clinically meaningful in CXRs and flipping can produce anatomically implausible images. Future work will systematically evaluate higher-resolution inputs, multi-crop/tiling strategies, and augmentation ablations to determine their potential to improve TB detection.

## 5. Conclusions

Our study investigated the development of a deep learning model for the detection of tuberculosis using CXRs. Our model, trained and internally validated with data from hospital A, achieved an area under the curve (AUC) of 0.915 and an AUC of 0.850 in the external validation using data from hospital B. These findings suggest that our deep learning model (DLM) holds promise as a supplementary tool for TB screening, enabling earlier identification of potential TB cases and expediting diagnostic confirmation. Importantly, the model’s performance was superior when all CXRs were included compared to analysis restricted to CXRs with paired polymerase chain reaction (PCR) results. This observation highlights the potential impact of non-TB lesions mimicking TB on CXR, emphasizing the need for careful interpretation of model predictions. Further research is crucial to refine the model’s generalizability and transparency, solidifying its role in clinical practice. Future directions involve integrating our DLM with PCR testing, where AI-predicted TB-positive cases would be subjected to confirmatory PCR, potentially augmenting the overall accuracy of TB diagnosis.

## Figures and Tables

**Table 1:** Patient characteristics in the training, tuning, internal validation, and external validation sets.

Variables	Training Set	Tuning Set	Internal Validation Set	External Validation Set	*p*-Value
**Gender**					<0.001
Female	76,248 (47.7%)	30,531 (47.8%)	45,717 (48.9%)	38,773 (49.9%)	
Male	83,534 (52.3%)	33,393 (52.2%)	47,695 (51.1%)	38,863 (50.1%)	
**Tuberculosis**	89 (0.1%)	32 (0.1%)	50 (0.1%)	35 (0.0%)	0.749
**CXR (department)**					<0.001
ER	38,149 (23.9%)	15,495 (24.2%)	22,294 (23.9%)	18,737 (24.1%)	
IPD	50,312 (31.5%)	20,217 (31.6%)	30,016 (32.1%)	18,839 (24.3%)	
OPD	71,321 (44.6%)	28,212 (44.1%)	41,102 (44.0%)	40,060 (51.6%)	
**CXR (modality)**					<0.001
CR	43,092 (27.0%)	16,988 (26.6%)	25,333 (27.1%)	23,321 (30.0%)	
DX	116,690 (73.0%)	46,936 (73.4%)	68,079 (72.9%)	54,315 (70.0%)	
**CXR (view)**					<0.001
AP	8882 (5.6%)	3520 (5.5%)	5227 (5.6%)	3580 (4.6%)	
PA	150,900 (94.4%)	60,404 (94.5%)	88,185 (94.4%)	74,056 (95.4%)	
**Age (years)**	50.6 ± 18.9	50.7 ± 18.9	51.1 ± 18.8	53.4 ± 20.1	<0.001
**Disease history**					
DM	15,145 (9.5%)	6040 (9.4%)	8884 (9.5%)	12,260 (15.8%)	<0.001
HTN	3106 (1.9%)	1198 (1.9%)	1827 (2.0%)	3069 (4.0%)	<0.001
HLP	20,919 (13.1%)	8403 (13.1%)	11,976 (12.8%)	20,102 (25.9%)	<0.001
CKD	9097 (5.7%)	3734 (5.8%)	5378 (5.8%)	6052 (7.8%)	<0.001
HF	4217 (2.6%)	1703 (2.7%)	2441 (2.6%)	3783 (4.9%)	<0.001
CAD	12,782 (8.0%)	5227 (8.2%)	7551 (8.1%)	10,709 (13.8%)	<0.001
COPD	10,942 (6.8%)	4351 (6.8%)	6294 (6.7%)	11,499 (14.8%)	<0.001

ER, Emergency Room; IPD, In-Patient Department; OPD, Out-Patient Department; CR, Computed Radiography; DX, Digital Radiography; AP, Anteroposterior; PA, Posteroanterior; DM, Diabetes Mellitus; HTN, Hypertension; HLP, Hyperlipidemia; CKD, Chronic Kidney Disease; HF, Heart Failure; CAD, Coronary Artery Disease; COPD, Chronic Obstructive Pulmonary Disease.

**Table 2:** The demographic characteristics, stratified by tuberculosis diagnosis in the internal and external validation sets.

	Internal Validation			External Validation		
Variables	Tuberculosis	Non-Tuberculosis	*p*-Value	Tuberculosis	Non-Tuberculosis	*p*-Value
**Gender**			0.003			0.863
Female	14 (28.0%)	45,681 (49.0%)		18 (51.4%)	38,734 (50.0%)	
Male	36 (72.0%)	47,592 (51.0%)		17 (48.6%)	38,785 (50.0%)	
**CXR (department)**			<0.001			<0.001
ER	7 (14.0%)	22,274 (23.9%)		3 (8.6%)	18,724 (24.2%)	
IPD	36 (72.0%)	29,945 (32.1%)		21 (60.0%)	18,787 (24.2%)	
OPD	7 (14.0%)	41,054 (44.0%)		11 (31.4%)	40,008 (51.6%)	
**CXR (modality)**			0.274			0.096
CR	17 (34.0%)	25,290 (27.1%)		6 (17.1%)	23,297 (30.1%)	
DX	33 (66.0%)	67,983 (72.9%)		29 (82.9%)	54,222 (69.9%)	
**CXR (view)**			<0.001			<0.001
AP	15 (30.0%)	5194 (5.6%)		10 (28.6%)	3558 (4.6%)	
PA	35 (70.0%)	88,079 (94.4%)		25 (71.4%)	73,961 (95.4%)	
**Age (years)**	68.1 ± 19.1	51.0 ± 18.8	<0.001	61.0 ± 21.7	53.4 ± 20.1	0.025
**Disease history**						
DM	13 (26.0%)	8844 (9.5%)	0.001	7 (20.0%)	12,232 (15.8%)	0.493
HTN	4 (8.0%)	1820 (2.0%)	0.016	2 (5.7%)	3060 (3.9%)	0.649
HLP	12 (24.0%)	11,940 (12.8%)	0.018	9 (25.7%)	20,067 (25.9%)	0.981
CKD	18 (36.0%)	5337 (5.7%)	<0.001	9 (25.7%)	6017 (7.8%)	0.001
HF	4 (8.0%)	2429 (2.6%)	0.041	3 (8.6%)	3769 (4.9%)	0.241
CAD	7 (14.0%)	7530 (8.1%)	0.121	5 (14.3%)	10,691 (13.8%)	0.810
COPD	9 (18.0%)	6260 (6.7%)	0.006	7 (20.0%)	11,473 (14.8%)	0.286

ER, Emergency Room; IPD, In-Patient Department; OPD, Out-Patient Department; CR, Computed Radiography; DX, Digital Radiography; AP, Anteroposterior; PA, Posteroanterior; DM, Diabetes Mellitus; HTN, Hypertension; HLP, Hyperlipidemia; CKD, Chronic Kidney Disease; HF, Heart Failure; CAD, Coronary Artery Disease; COPD, Chronic Obstructive Pulmonary Disease.

## Data Availability

Research data is unavailable due to privacy or ethical restrictions.

## Supplementary Materials

The following supporting information can be downloaded at https://www.mdpi.com/article/10.3390/bioengineering12121377/s1. Table S1: DeLong’s test comparing internal and external AUCs across subgroups; Table S2: DeLong’s test comparing internal and external AUCs across subgroups (PCR only).

## Abbreviations

The following abbreviations are used in this manuscript:

AI	Artificial intelligence
TB	Tuberculosis
CXR	Chest X-ray
PCR	Polymerase chain reaction
DLM	Deep learning model
ROC	Receiver operating characteristic
AUC	Area under the curve
ER	Emergency room
IPD	In-patient department
OPD	Out-patient department
CR	Computed radiography
DX	Digital radiography
AP	Anteroposterior
PA	Posteroanterior
DM	Diabetes mellitus
HTN	Hypertension
HLP	Hyperlipidemia
CKD	Chronic kidney disease
HF	Heart failure
CAD	Coronary artery disease
COPD	Chronic obstructive pulmonary disease
