# Supplementary material for: Integrating AI with PCR for Tuberculosis Diagnosis: Evaluating a Deep Learning Model for Chest X-Rays
Source: Bioengineering (Basel). 2025 Dec 18;12(12):1377. doi: 10.3390/bioengineering12121377 (PMC12730101; doi:10.3390/bioengineering12121377)
Supplement: Supplementary file 1 [file bioengineering-12-01377-s001.zip › bioengineering-3955751-supplementary.pdf]

Supplementary Table S1. DeLong's test comparing internal and external AUCs across subgroups.

Group	Tuberculosis						Delong's p-value
	Internal			External			
	AUC	Lower	Upper	AUC	Lower	Upper	
**all samples**	0.92	0.86	0.97	0.85	0.78	0.92	0.153
**CXR (view)**							
AP	0.84	0.75	0.93	0.74	0.55	0.93	0.350
PA	0.91	0.84	0.98	0.85	0.76	0.94	0.308
**CXR (modality)**							
CR	0.89	0.78	1.00	0.87	0.67	1.00	0.870
DX	0.93	0.87	0.98	0.84	0.75	0.92	0.072
**CXR (department)**							
ER	0.89	0.78	1.00	0.81	0.45	1.00	0.672
IPD	0.93	0.88	0.97	0.82	0.71	0.93	0.076
OPD	0.81	0.55	1.00	0.86	0.74	0.98	0.748
**Gender**							
Female	0.87	0.76	0.98	0.79	0.66	0.91	0.337
Male	0.93	0.87	0.99	0.91	0.85	0.98	0.723
**Age**							
<55 y/o	0.91	0.75	1.00	0.87	0.74	0.99	0.685
55-64 y/o	0.89	0.70	1.00	0.90	0.72	1.00	0.961
65-74 y/o	0.92	0.85	0.99	0.85	0.59	1.00	0.615
≧75 y/o	0.81	0.72	0.90	0.69	0.53	0.85	0.201
**Gender-Age**							
Female <55 y/o	0.80	0.44	1.00	0.73	0.50	0.95	0.716
Male <55 y/o	0.99	0.96	1.00	0.99	0.98	1.00	0.626
Female 55-64 y/o	-	-	-	0.98	0.97	1.00	-
Male 55-64 y/o	0.88	0.66	1.00	0.80	0.43	1.00	0.741
Female 65-74 y/o	0.87	0.63	1.00	0.87	0.64	1.00	0.989
Male 65-74 y/o	0.92	0.87	0.98	0.63	0.30	0.95	0.077
Female ≧75 y/o	0.76	0.58	0.93	0.71	0.55	0.87	0.693
Male ≧75 y/o	0.81	0.71	0.92	0.83	0.74	0.92	0.794
**Disease history**							
Without DM	0.91	0.85	0.98	0.95	0.91	0.99	0.322
With DM	0.91	0.82	0.99	0.85	0.77	0.92	0.320
Without HTN	0.93	0.90	0.97	0.88	0.75	1.00	0.508
With HTN	0.67	0.23	1.00	0.83	0.74	0.92	0.477
Without HLP	0.92	0.85	0.98	0.91	0.80	1.00	0.944
With HLP	0.89	0.82	0.97	0.84	0.76	0.93	0.396
Without CKD	0.94	0.89	0.99	0.80	0.62	0.98	0.149
With CKD	0.78	0.66	0.91	0.84	0.75	0.92	0.491
Without CAD	0.93	0.89	0.97	0.96	0.90	1.00	0.410

With CAD	0.82	0.55	1.00	0.86	0.78	0.93	0.785
Without HF	0.92	0.86	0.97	0.72	0.25	1.00	0.419
With HF	0.82	0.67	0.97	0.86	0.77	0.94	0.669
Without COPD	0.91	0.85	0.97	0.81	0.64	0.98	0.242
With COPD	0.89	0.79	0.98	0.61	0.50	0.72	0.000

ER, Emergency Room; IPD, In-Patient Department; OPD, Out-Patient Department; CR, Computed Radiography; DX, Digital Radiography; AP, Anteroposterior; PA, Posteroanterior; DM, Diabetes Mellitus; HTN, Hypertension; HLP, Hyperlipidemia; CKD, Chronic Kidney Disease; HF, Heart Failure; CAD, Coronary Artery Disease; COPD, Chronic Obstructive Pulmonary Disease

Supplementary Table S2. DeLong's test comparing internal and external AUCs across subgroups (PCR only).

	Tuberculosis (PCR only)						
	Internal			External			
**Group**	**AUC**	**Lower**	**Upper**	**AUC**	**Lower**	**Upper**	**Delong's p-value**
**all samples**	0.73	0.66	0.81	0.60	0.38	0.81	0.232
**CXR (view)**							
AP	0.72	0.58	0.86	0.62	0.50	0.75	0.306
PA	0.74	0.65	0.83	0.63	0.38	0.88	0.422
**CXR (modality)**							
CR	0.69	0.54	0.84	0.60	0.49	0.72	0.360
DX	0.75	0.67	0.84	0.71	0.21	1.00	0.857
**CXR (department)**							
ER	0.75	0.54	0.96	0.61	0.47	0.75	0.255
IPD	0.77	0.69	0.85	0.63	0.45	0.82	0.177
OPD	0.59	0.34	0.83	0.56	0.40	0.72	0.874
**Gender**							
Female	0.67	0.51	0.84	0.67	0.53	0.82	0.998
Male	0.74	0.66	0.83	0.73	0.56	0.90	0.879
**Age**							
<55 y/o	0.79	0.60	0.99	0.75	0.49	1.00	0.817
55-64 y/o	0.82	0.63	1.00	0.62	0.19	1.00	0.412
65-74 y/o	0.71	0.53	0.88	0.56	0.38	0.73	0.239
≧75 y/o	0.64	0.51	0.76	0.44	0.16	0.72	0.208
**Gender-Age**							
Female <55 y/o	0.71	0.32	1.00	0.90	0.79	1.00	0.382
Male <55 y/o	0.87	0.71	1.00	0.89	0.78	1.00	0.806
Female 55-64 y/o	-	-	-	0.66	0.09	1.00	-
Male 55-64 y/o	0.80	0.59	1.00	0.64	0.23	1.00	0.500
Female 65-74 y/o	0.70	0.14	1.00	0.58	0.27	0.89	0.711
Male 65-74 y/o	0.69	0.51	0.86	0.56	0.31	0.80	0.398
Female ≧75 y/o	0.43	0.16	0.70	0.57	0.45	0.69	0.368

Male ≧75 y/o	0.64	0.50	0.79	0.80	0.64	0.96	0.147
**Disease history**							
Without DM	0.72	0.64	0.81	0.61	0.50	0.72	0.112
With DM	0.77	0.60	0.93	0.58	0.17	0.99	0.422
Without HTN	0.76	0.68	0.83	0.57	0.44	0.70	0.013
With HTN	0.57	0.26	0.88	0.74	0.55	0.92	0.356
Without HLP	0.76	0.67	0.84	0.61	0.49	0.73	0.060
With HLP	0.68	0.52	0.84	0.63	0.40	0.86	0.731
Without CKD	0.78	0.70	0.87	0.58	0.47	0.70	0.007
With CKD	0.64	0.50	0.79	0.82	0.60	1.00	0.193
Without CAD	0.74	0.66	0.82	0.61	0.50	0.72	0.056
With CAD	0.71	0.44	0.98	0.59	0.03	1.00	0.722
Without HF	0.74	0.66	0.82	0.63	0.51	0.75	0.148
With HF	0.66	0.40	0.92	0.52	0.30	0.75	0.431
Without COPD	0.74	0.66	0.83	0.64	0.23	1.00	0.647
With COPD	0.68	0.49	0.88	0.87	0.64	1.00	0.219

ER, Emergency Room; IPD, In-Patient Department; OPD, Out-Patient Department; CR, Computed Radiography; DX, Digital Radiography; AP, Anteroposterior; PA, Posteroanterior; DM, Diabetes Mellitus; HTN, Hypertension; HLP, Hyperlipidemia; CKD, Chronic Kidney Disease; HF, Heart Failure; CAD, Coronary Artery Disease; COPD, Chronic Obstructive Pulmonary Disease
